# Perspective: Pathological transdifferentiation—a novel therapeutic target for cardiovascular diseases and chronic inflammation

**DOI:** 10.3389/fcvm.2024.1500775

**Published:** 2024-11-26

**Authors:** William Y. Yang, Mohammed Ben Issa, Fatma Saaoud, Keman Xu, Ying Shao, Yifan Lu, Waleska Dornas, Ramon Cueto, Xiaohua Jiang, Hong Wang, Xiaofeng Yang

**Affiliations:** ^1^ Department of Cardiovascular Sciences, Lemole Center for Integrated Lymphatics and Vascular Research, Lewis Katz School of Medicine at Temple University, Philadelphia, PA, United States; ^2^ Department of Biochemistry and Immunology, Institute of Biological Sciences, Federal University of Minas Gerais, Belo Horizonte, Brazil; ^3^ Department of Cardiovascular Sciences, Metabolic Disease Research and Thrombosis Research Center, Lewis Katz School of Medicine at Temple University, Philadelphia, PA, United States

**Keywords:** pathological transdifferentiation, chronic inflammation, cardiovascular diseases, epithelial to mesenchymal transition, endothelial to mesenchymal transition, vascular smooth muscle to mesenchymal transition

## Abstract

Pathological transdifferentiation, where differentiated cells aberrantly transform into other cell types that exacerbate disease rather than promote healing, represents a novel and significant concept. This perspective discusses its role and potential targeting in cardiovascular diseases and chronic inflammation. Current therapies mainly focus on mitigating early inflammatory response through proinflammatory cytokines and pathways targeting, including corticosteroids, TNF-α inhibitors, IL-1β monoclonal antibodies and blockers, IL-6 blockers, and nonsteroidal anti-inflammatory drugs (NSAIDs), along with modulating innate immune memory (trained immunity). However, these approaches often fail to address long-term tissue damage and functional regeneration. For instance, fibroblasts can transdifferentiate into myofibroblasts in cardiac fibrosis, and endothelial cells may undergo endothelial to mesenchymal transition (EndMT) in vascular remodeling, resulting in fibrosis and impaired tissue function. Targeting pathological transdifferentiation represents a promising therapeutic avenue by focusing on key signaling pathways that drive these aberrant cellular phenotypic and transcriptomic transitions. This approach seeks to inhibit these pathways or modulate cellular plasticity to promote effective tissue regeneration and prevent fibrosis. Such strategies have the potential to address inflammation, cell death, and the resulting tissue damage, providing a more comprehensive and sustainable treatment solution. Future research should focus on understanding the mechanisms behind pathological transdifferentiation, identifying relevant biomarkers and master regulators, and developing novel therapies through preclinical and clinical trials. Integrating these new therapies with existing anti-inflammatory treatments could enhance efficacy and improve patient outcomes. Highlighting pathological transdifferentiation as a therapeutic target could transform treatment paradigms, leading to better management and functional recovery of cardiovascular tissues in diseases and chronic inflammation.

## Introduction

Chronic inflammation, while contributing to many chronic and degenerative diseases as pathological process, it also plays a role in mitigating infections, clearing damaged cells, and initiating tissue repair. Recent advances have provided insights into the cellular and molecular mechanisms by which traditional inflammatory cytokines (1) and Wnt/b-catenin factors (2, 3) control tissue repair and regeneration, suggesting that reparative inflammation drives tissue regeneration (4). It has long been recognized that chronic tissue damages leads to structural destruction, cell death, and fibrosis, as seen in occupational respiratory diseases like coal workers' pneumoconiosis and silicosis (5), pulmonary artery erosion, Rasmussen aneurysm (6), extensive scarring in the upper lobes of the lungs from tuberculosis (7), burn scars (8), and myocardial scaring after myocardial infarction (9).

Increasing tissue differentiation in damaged tissue has been considered a therapeutic approach for tissue regeneration. In the differentiation process, a pluripotent stem cell undergoes proliferation and progresses through intermediate and progenitor stages before losing its multipotent capacities (10, 11) and differentiating into specialized, mature cells that form organs or tissues (10, 12). Simple combinations of lineage-determining transcription factors (TFs) activate the cis-regulatory elements necessary for establishing macrophage and B cell identities (13). While the B cell lineage is highly resistant to most differentiation factors, the loss of a single B lineage-specific TF or the enforced expression of TFs from non-B cell lineages can interfere with B cell maintenance, allowing for dedifferentiation or trans-differentiation into other different lineages (14). Promoters driven by lineage-determining TF control the expression of macrophage-specific genes in a cell type dependent manner (15). Ng A. et al. introduced the Human TFome, an extensive library containing 1,564 TF genes and 1,732 TF splice isoforms. By screening this library across three human pluripotent stem cell (hPSC) lines, they identified 290 TFs, 241 of which were previously unknown, that trigger differentiation within just four days, without changing external soluble factors or biomechanical signals (16). The temporal regulation of lineage-specific TFs driven by super-enhancer (SE) is fundamental to normal developmental programs (17). Two additional processes related to differentiation are de-differentiation, where a differentiated cell returns to a less-differentiated state to regain its proliferative capacity, and trans-differentiation, where one differentiated cell type converts directly into another without returning to a pluripotent state. As a result, transdifferentiation is commonly referred to as direct cell reprogramming (18). Both dedifferentiation and transdifferentiation can occur spontaneously in natural biological processes (19).

Research has shown that pathological transdifferentiation is often associated with the development of cardiovascular diseases. For example, after myocardial infarction (MI), adult cardiomyocytes have a limited ability to regenerate. As a result, cardiac fibroblasts (CFs) derived from resident and/or other cell types produce the extracellular matrix, resulting in fibrosis, which contributes to cardiac dysfunction and heart failure (20). As Dr. Fu et al. highlighted, the signaling pathways that regulate the various differentiation potentials of cardiac fibroblasts may warrant greater focus, given the growing evidence of these differentiations in diseased hearts (21). In addition, while the cause of aortic aneurysms is not well understood, it is linked to hypercholesterolemia, atherosclerosis, and abnormal signaling of transforming growth factor-β (TGF-β) in vascular smooth muscle cells (VSMCs) (22). This combination of medial VSMC loss with increased non-VSMC aortic cell mass results in excessive growth and dilation of the aorta, along with calcification and ossification of the aortic wall, and inflammation, ultimately leading to aneurysm formation (1, 22–24).

Epithelial to mesenchymal transition (EMT) is a biological process in which epithelial cells shed their defining characteristics, such as tight junctions and polarity, and gain mesenchymal properties, like increased motility and invasiveness (25). This transition is crucial in various physiological processes, including embryogenesis, wound healing (26), tissue fibrosis, and cancer (27, 28). As our knowledge of EMT regulation grows, it is becoming increasingly apparent that EMT exists on a spectrum, with a range of intermediate cellular states between the fully epithelial and fully mesenchymal phenotypes. Rather than existing in distinct, “pure” epithelial or mesenchymal forms, cells can adopt intermediate or hybrid states, exhibiting a combination of traits from both epithelial and mesenchymal characteristics indicating that cells undergo partial EMT (29–31). In the context of cardiovascular diseases, EMT is a crucial factor in various pathological conditions (32), including atherosclerosis, vascular remodeling, cardiac fibrosis, heart valve diseases, and myocardial infarction, involving different signaling pathways and molecular mechanisms such as TGF-β, Notch signaling, Wnt/β-catenin, and inflammatory cytokines (33–35). Endothelial to mesenchymal transition (EndMT) is the process through which endothelial cells lose their specific markers and acquire mesenchymal phenotypes, potentially through metabolic reprogramming (36). This underlies regulatory networks involved in cardiovascular disease (CVD) (37), pulmonary vascular diseases (38), fibrotic disorders (39, 40) such as pulmonary fibrosis (41), and ischemic stroke (42). In addition to a complete EndMT, recent studies indicate that ECs can undergo a partial or incomplete EndMT, leading to the transient and reversible formation of intermediate cells that display a combination of both endothelial and mesenchymal features (40). This partial EndMT allows for flexible cellular states process is transient and reversible, playing a crucial role in developmental angiogenesis, pathological vascular remodeling. The ability of ECs to adopt this intermediate state allows for flexible adaptation in response to various physiological and pathological conditions, contributing to tissue regeneration, wound healing, and disease progression in atherosclerotic cardiovascular diseases (CVDs), metabolic CVDs, vascular fibrosis, and cancer (40, 43). In patients with acute coronary syndrome, thrombus leukocytes show a higher expression of endothelial cell-specific angiogenic markers compared to peripheral blood leukocytes, suggesting transdifferentiation from leukocytes into angiogenic endothelial cells (44). Under the influence of proinflammatory cytokines like tumor necrosis factor (TNF)-α and CD4^+^ T helper cell 1 (Th1) cytokine interferon-γ (IFNγ) (45), human liver sinusoidal endothelial cells upregulate six immune checkpoints (46) and transdifferentiate into immune tolerogenic cells (47).

In this prospective, we propose pathological transdifferentiation as a new concept and novel therapeutic target for functional tissue regeneration following tissue damage related to cardiovascular diseases and chronic inflammation. Unlike current therapies targeting the proinflammatory cytokine-driven early phase of cardiovascular inflammation and innate immune memory (also known as trained immunity 48–50), our focus here is on targeting pathological transdifferentiation after tissue damage. Further understanding of pathological transdifferentiation in cardiovascular diseases and chronic inflammation will significantly advance the development of new therapies for these conditions.

## Pathological transdifferentiation: a key contributor to weakened tissue function in cardiovascular diseases and chronic inflammation

Impaired tissue function in cardiovascular diseases results from persistent cellular damage and maladaptive responses that disturb normal tissue homeostasis and weaken physiological functions (51). In cardiovascular diseases, factors such as atherosclerosis, ischemia, diabetes, and metabolic disorders can lead to endothelial cell dysfunction, fibrosis, and vascular remodeling, impairing blood vessel function and contributing to heart failure (52). Chronic inflammation, driven by immune system dysregulation, leads to prolonged activation of inflammatory pathways, causing tissue damage, pathological cell transdifferentiation, and the loss of regenerative capacity (53). To improve our understanding of chronic cardiovascular inflammation, we and others have demonstrated the following: (1) Endogenous metabolite-derived conditional danger-associated molecular patterns (54, 55), such as lysophospholipids (56), trigger the innate immune transdifferentiation of endothelial cells, leading to sustained endothelial activation (57–59). This supports our working model that endothelial cells, angiogenic endothelial cells (60), and angiogenic progenitor cells (61, 62) function as innate immune cells (50, 58, 59); (2) Similar to other CVD risk factors, including hyperlipidemia, diabetes (63), obesity (64–66), hypertension, and cigarette smoke (67), Dr. Wang's team reported that severe hyperhomocysteinemia (HHcy) (68) promotes the differentiation of both bone marrow–derived and resident inflammatory monocytes, which in turn accelerates the development of atherosclerosis in mice lacking low-density lipoprotein receptor (LDLR) and cystathionine β-synthase (CBS) (69–71). Additionally, advanced single-cell analysis of human peripheral blood mononuclear cells (72) reveals distinct intermediate monocyte subsets that correlate with gender variation in coronary artery disease (73); (3) Pathological conditions re-shape physiological CD4+ Foxp3+ regulatory T cells (Tregs) (74) into pathological Tregs (75). We have extensively published data supporting this concept (11, 76–78). Transcription factors such as ATA binding protein 3 (GATA3), BCL6 transcription repressor (BCL6), and histone deacetylase 6 (HDAC6) regulate Treg trandifferentiation (11, 74–77) into antigen-presenting cell (APC)-like Tregs or Th1-Tregs (45). Additionally, immune checkpoints regulate T cell and Treg transdifferentiation and immune tolerance (46, 47, 79). Cigarette smoke combined with morphine reprograms Treg transcriptomes, transdifferentiating anti-inflammatory/immunosuppressive Tregs into proinflammatory/pathogenic CD4^+^ T helper cell 17 (Th17) cells (80–82). Moreover, 67 different Treg subsets have been identified in human diseases, with 44 subsets identified in cancers including 7 Treg subsets in colorectal cancer, 10 Treg subsets in lung cancer, 9 Treg subsets in liver cancer, 4 Treg subsets in gastric cancer, 2 Treg subsets in breast cancer, 3 Treg subsets in neck carcinoma, 1 Treg subset in cutaneous carcinoma, 2 Treg subsets in cervical cancer, 2 Treg subsets in ovarian cancer, 2 Treg subsets in melanoma, 1 Treg subset in bladder cancer, and 1 Treg subset in follicular lymphoma (77, 83). In addition, 23 different subsets of Treg have been identified in relation to human autoimmune diseases (84), transplantation, and pregnancy, including 4 Treg subsets in systemic lupus erythematosus, 4 Treg subsets in multiple sclerosis, 3 Treg subsets in rheumatoid arthritis, 2 Treg subsets in type 1 diabetes, 2 Treg subsets in organ transplantation, 5 Treg subsets in graft-vs.-host disease, and 3 Treg subsets in pregnancy (11, 85); (4) The aorta, under pathological conditions, functions as an immune organ, upregulating secretomes and providing a microenvironment that support the activation, differentiation, and trans-differentiation of immune and vascular cells. In the early stages, these secretomes may play a pivotal role in driving trained immunity (innate immune memory for inflammation enhancement) (1), as we recently identified IL-1β as a second-tier driver of trained immunity (86); (5) VSMCs have previously been categorized into two types: contractile and de-differentiated (synthetic), characterized by the upregulation of cell proliferation genes (87). However, research using single-cell RNA sequencing (scRNA-Seq) has shown that de-differentiated arterial VSMCs exhibit significant diversity, including mesenchymal (we propose to newly term VSMC to mesenchymal transition here), fibroblast, myofibroblast, osteogenic, osteochondrogenic, macrophage (88), foam cell, adipocyte, and mesenchymal stem cell-like phenotypes (89, 90). Currently, there is no consensus on the numbers of VSMC phenotypes or transcriptomic types (91). Using knowledge-based transcriptomic analysis (92), we reported that the innate immune function of VSMCs plays a role in two-wave inflammation associated with atherosclerosis and twin-peak inflammation seen in aortic aneurysms, and their potential to trans-differentiate into 25 distinct cell types (24); (6) Angiotensin II (Ang II) was found to induce abdominal aortic aneurysm (AAA) and thoracic aortic aneurysm (TAA) in apolipoprotein E-deficient (ApoE^−/−^) hyperlipidemic mice, leading to the upregulation of 73 and 68 cytokines, respectively. scRNA-Seq identified specific markers associated with macrophages, immune cells, regulators of cell death, and transdifferentiation indicators of neuronal, glial, and squamous epithelial cells in different segments of the aortic, demonstrating that cell transdifferentiation varies across different aortic pathologies (22); (7) Contrary to the traditional model of proinflammatory M1 macrophages and anti-inflammatory M2 macrophages, 32 macrophage pathways/subsets. This includes 20 novel disease-specific pathways and 12 new shared pathways, which has been observed across eight groups of 34 diseases, encompassing 24 inflammatory organ diseases and 10 different types of tumors (93); (8) Chronic kidney disease (72) has been shown to transdifferentiates veins into specialized immune-endocrine organs, characterized by increased signaling of the MYCN proto-oncogene and BHLH transcription factor (MYCN)- activator protein 1 (AP1) signaling (94); and (9) Dr. Vazquez-Padron's team reported that arteries and veins develop distinct types of occlusive diseases and exhibit different responses to injury. Cellular atlases from six veins and arteries reveal that arteries have a 7.8-fold higher proportion of contractile VSMCs and trend to show more modulated VSMCs. In contrast, veins have a greater abundance of endothelial cells, macrophages, and pericytes, along with a rising trend in fibroblast numbers. While activated fibroblasts are found in similar proportions in both vessel types, there are significant differences in their gene expression. Modulated VSMCs (87) and activated fibroblasts are marked by the upregulation of genes such as myosin heavy chain 10 (MYH10), Fibronectin 1 (FN1), collagen type VIII alpha 1 chain (COL8A1), and integrin subunit alpha 10 (ITGA10). Activated fibroblasts also express coagulation factor II thrombin receptor (F2R), periostin (POSTN), and cartilage oligomeric matrix protein (COMP), which have been confirmed through F2R/CD90 flow cytometry. Among fibroblast populations from both arteries and veins, activated fibroblasts from veins are the highest producers of collagens. These venous fibroblasts also exhibit high levels of angiogenesis, proinflammatory activity, and a heightened response to reactive oxygen species (ROS) (95). In arteries, fibroblasts are predominantly located outside the external elastic lamina, whereas in veins, they are more widely distributed throughout the entire venous wall. Consistent with this observation, extracellular matrix (ECM)-targeted proteomics has revealed a greater abundance of fibrillar collagens in veins, while arteries show a higher concentration of basement membrane ECM components (96). However, molecular mechanisms underlying chronic inflammation-induced transdifferentiation remain poorly characterized.

## Pathological transdifferentiation pathways are new therapeutic targets

Cancer heterogeneity, rather than oligomeric or clonal expansion of cancer cells, has emerged as a key factor influencing therapy outcomes. As a result, many new cancer treatments now target these heterogeneous cell populations. Although short-term goals have been achieved with treatments like chemotherapy, radiotherapy, anti-angiogenesis therapy, and immunotherapy, long-term cancer regression remains challenging. Consequently, researchers are exploring cellular reprogramming in cancer, suggesting that cancer is a dynamic process rather than a static cellular state, as highlighted by the classic hallmark of cancer (97). Neuroblastoma (NB), for example, arises from the failure of sympathoadrenal progenitor cells to exit their self-renewal phase. Banerjee, D. et al. demonstrated that all-trans retinoic acid (ATRA) can induce growth arrest and differentiation in NB cells, identifying super-enhancers (SEs) that drive TF regulators (24). Time-course histone 3 lysine 27 acetylation (H3K27ac) (98) chromatin immunoprecipitation-DNA sequencing (ChIP-Seq) (99) and RNA-Seq revealed ATRA-coordinated SE waves. CRISPR-Cas9 and siRNA experiments further confirmed that the stem cell development TFs, including SOX11, MYCN, and GATA3, are crucial for this process both *in vitro* and *in vivo*. Additional TFs involved in neural development, such as GATA2 and SOX4, were activated in distinct waves at 2, 4, and 8 days of ATRA treatment, identifying oncogenic lineage drivers that sustain NB self-renewal and are essential for the differentiation process (17).

Moreover, chronic inflammation driven by immune dysregulation plays a key role in promoting tumor development. The inflammatory microenvironment influences the fate of lymphocytes through mediators such as cytokines and chemokines. For example, tumor cells secrete immunosuppressive cytokines like vascular endothelial growth factor (VEGF) and TGF-β (100), which facilitate the conversion of effector T cells into CD4^+^ Foxp3^+^ Tregs. These Tregs then secrete anti-inflammatory cytokines, including IL-10 and IL-35 (76, 99–102), as well as exosomes (74) and express two prototypic immune checkpoints (46, 47, 77) such as cytotoxic T-lymphocyte associated protein 4 (CTLA4) and programed cell death 1 ligand 1 (PD-L1), suppressing cytotoxic T lymphocyte (CTL) functions (46, 47, 77, 103, 104).

A recent report using single-nucleus RNA-sequencing (snRNA-Seq) on forty-seven liver biopsies collected from patients at various stages of metabolic dysfunction-associated steatotic liver disease established a detailed cellular map of liver changes throughout disease progression. Combining this single-cell data with advanced 3D imaging techniques, Gribben C. et al. revealed profound changes in liver architecture, including the loss of hepatocytes zonation and significant reorganization of the biliary tree. Remarkably, transdifferentiation events between hepatocytes and cholangiocytes were observed, occurring independently of adult stem cells or the activation of developmental progenitors (105). Functional analyses of cholangiocyte organoids confirmed that the PI3K–protein kinase B (AKT)– mammalian target of rapamycin (mTOR) pathway plays a critical role in plasticity and its connection to insulin signaling (106). These findings demonstrate that chronic diseases and injuries induce cellular plasticity in human organs, potentially opening new therapeutic avenues for managing chronic diseases (107).

Cell transdifferentiation and reprogramming have opened possibilities for manipulating cell fate through various external, pharmacological, and therapeutic interventions. Small molecules and elements of the transcription regulatory machinery are emerging as valuable tools for executing cell transdifferentiation, providing new opportunities for disease modeling and drug discovery (108). At more advanced stages, these methods could lead to the development of more efficient and precise gene therapies, as well as enhanced regenerative medicine approaches (109). To address the challenge of detecting low or absent expression of many disease-related genes/transcripts in clinically accessible samples, Li, S., and colleagues devised a diagnostic method that enhances the identification and analysis of tissue-specific genes/transcripts by employing fibroblast to neuron transdifferentiation. They generated induced neurons (iNeurons) from seventy-one patients with primary neurological symptoms enrolled in the Undiagnosed Diseases Network, achieving a 25.4% diagnostic success rate. More than a quarter of these diagnoses were made possibly by transdifferentiation, which would have been missed using fibroblast RNA-seq alone. This iNeuron transdifferentiation-transcriptomic approach can be seamlessly incorporated into whole transcriptome evaluations for genetic disorders (110). In 2015, Fu, Y. and collaborators announced the successful generation of spontaneously beating cardiomyocyte-like cells derived from mouse fibroblasts, achieved solely using chemical cocktails. These chemically induced cardiomyocyte-like cells (CiCMs) expressed markers specific to cardiomyocytes, showed organized sarcomeric structures, and displayed typical cardiac calcium flux and electrophysiological characteristic. Genetic lineage tracing confirmed that these CiCMs originated from fibroblasts. Furthermore, the generation of CiCMs involved a cardiac progenitor stage rather than reverting to a pluripotent state. This discovery, which eliminate the need of viral factors, establishes a foundation for *in vivo* cardiac transdifferentiation using pharmacological agents, presenting a potentially safer approach for treating heart failure (111). In addition, advanced cell and gene therapies in cardiology are among the new therapies for pathological transdifferentiation (112).

## Role of reactive oxygen species (ROS) in pathological transdifferentiation: impact of stimuli intensity, cellular context, and metabolic status

Reactive oxygen species (ROS) are vital contributors to fundamental cellular functions, including cell proliferation, survival, and the sensing of metabolic reprogramming (95). Additionally, ROS play a key role in innate immunity in endothelial cells (58, 59, 113) and VSMCs (22, 24), as well as in stem cell (10) maintenance and neuronal differentiation. In a model of human embryonic stem cell (hESC) using Ntera2 (NT2) cells (114), assays using CM-H2DCFDA (a general indicator of oxidative stress) and dihydroethidium (DHE) verified that the oxidizing agent paraquat induces high levels of ROS in NT2 cells. Paraquat-induced oxidative stress suppresses the expression of stemness markers, such as POU class 5 homeobox 1 (POU5F1, also known as OCT4), nanog homeobox (NANOG), and Cripto (a member of the epidermal growth factor-Cripto-1/FRL-1/Cryptic (EGF-CFC) family, while enhancing the spontaneous expression of neuronal differentiation markers, including paired box 6 (PAX6), neuronal differentiation 1 (NEUROD1), homeobox A1 (HOXA1), neural cell adhesion molecule 1 (NCAM), GDNF family receptor alpha 1 (GFRA1) and class III beta-tubulin (TUJ1). Additionally, the treated cells displayed a markedly different morphology compared to the control cells, characterized by the extension of long neurite-like processes.

The neurogenic effect of ROS on stem cell behavior is suppressed by an antioxidant, but this effect is further intensified by the knockdown of NFE2 like BZIP transcription factor 2 (Nrf2), which is a crucial transcription factor involved in antioxidant signaling (113). Furthermore, paraquat activates the neurogenic mitogen-activated protein kinase (MAPK) pathways, specifically the extracellular signal-regulated kinase 1/2 (ERK1/2) pathways, in a dose-dependent manner (115, 116). This activation can be counteracted by the MEK1/2 inhibitor SL327. These findings indicate that excessive intracellular ROS can lead to a transition away from the stem cell state, promoting the neuronal differentiation of hESCs, with MAPK-ERK1/2 signaling playing a significant role in ROS-induced neuronal differentiation (114). Additionally, nicotinamide adenine dinucleotide phosphate (NADPH) oxidase 4 (NOX4) (117) generates ROS in CD34^+^ cells during the vascular differentiation of hESCs, suggesting that and modulation of ROS levels with antioxidants, such as selenium, could provide a novel strategy to enhance vascular differentiation efficiency in hESCs (118).

ROS have been linked to mechanisms of heart development and regenerative therapies, particularly in the context of pluripotent stem cell applications. The role of ROS in mediating cell fate depends on several factors, including the strength of stimuli, the cellular context, and the metabolic state of the cells. ROS exert their effects through various targets, such as kinases and transcription factors, playing diverse roles at distinct phases of cardiac differentiation, proliferation, and maturation. The detrimental effects of the host's ROS on graft (donor) cells have been reported in a paracrine manner during stem cell implantation. Therefore, it is crucial to carefully regulate the timing and levels of ROS production following myocardial injury to maximize the effectiveness of regenerative therapies while minimizing potential harm (119).

Recently, we investigated the role of innate immunity in VSMCs and related aortic pathologies through transcriptome analyses of aortas from proatherogenic apolipoprotein E deficient (Ape^−/−^) mice during both angiotensin II-induced aortic abdominal aneurysm (AAA) and an Ape^−/−^ atherosclerosis over time. Additionally, we analyzed VSMCs stimulated with conditional danger-associated molecular patterns (DAMPs) (54, 55). The DAMPs-stimulated VSMCs exhibited an enhanced potential for trans-differentiation, indicated by upregulation not only some of the 82 markers of seven VSMC-plastic cell types (91), including mesenchymal cells, macrophages, adipocytes, fibroblasts, myofibroblasts, osteogenic cells, and foam cells) but also markers of 18 novel cell types (from 79 human cell types with 8,065 markers identified in the Human Protein Atlas database https://www.proteinatlas.org/) (24). Further analysis of the transcriptomes from gene-deficient models revealed that the antioxidant transcription factor NRF2 plays a role in suppressing inflammation. In contrast, five other inflammatory transcription factors and master regulators —namely the aryl hydrocarbon receptor (AHR, which integrates environmental, dietary, microbial, and metabolic signals to control complex transcriptional programs in a ligand-specific, cell-type-specific and context-specific manner 120) (82), NF-KB, NADPH Oxidases (117) (NOX, enzyme responsible for ROS production), eukaryotic translation initiation factor 2 alpha kinase 3 [EIF2AK3, also known as PERK, an endoplasmic reticulum (ER) stress kinase] (121, 122), and SET domain containing 7 (SET7), a histone lysine methyltransferase associated with trained immunity (123) —were found to drive the upregulation of twelve lists of innate immune genes in the context of atherosclerosis, AAA, and DAMP-stimulated VSMCs (24).

## Metabolic reprogramming modulates cell-type specific gene expression through histone acylation

Liver fibrosis is characterized by the excessive and unbalanced accumulation of fibrous extracellular matrix (ECM) in response to hepatic wound healing. This is a common mechanism impairing liver function in many chronic liver diseases. Despite ongoing efforts, effective therapy for fibrosis have yet to be established. Alarmingly, the incidence of fibrosis is increasing as a result of the expanding obesity pandemic. In this review, we outlined the key components and mechanisms driving liver fibrosis, emphasizing the metabolic control of key fibrogenesis regulators, particularly hepatic stellate cells (HSCs), and their involvement in disease advancement. It underscores how the metabolic reprogramming of hepatic cells necessitates a finely tuned cellular response to fulfill energy demands without compromising cellular integrity. This review also highlights the role of RNA-binding proteins (RBPs), which, due to their context- and stimuli-dependent nature, are well-suited to respond to fibrotic conditions. This review also summarize current research on the metabolic regulation of HSCs in liver fibrosis and the role of RBPs in post-transcriptionally controlling the metabolic shift that drives fibrosis and chronic liver disease progression (124).

Chen, X et al. reviewed the manipulation of metabolic enzymes such as solute carrier family 2 member 1 (SLC2A1, Glut1), pyruvate kinase M1/2 (PKM, PKM2), pyruvate dehydrogenase kinase 4 (PDK4), lactate dehydrogenase A (LDHA), succinate dehydrogenase (SDH), carnitine palmitoyl transferase 1B (CPT1b), and 3-hydroxy-3-methylglutaryl-CoA synthase 2 (HMGCS2) to inhibit fatty acid oxidation or promote glycolysis, which is sufficient to promote cardiomyocyte proliferation, though not transdifferentiation (125). Cardiomyocytes possess distinct metabolic features that set them apart from cardiac fibroblasts and pluripotent stem cells (PSCs), with various overlapping molecular mechanisms driving metabolic reprogramming during cardiomyogenesis. Significant metabolic alterations observed during the differentiation of cardiomyocytes from PSCs and cardiac fibroblasts suggest that metabolic reprogramming may play a crucial role in promoting cardiomyogenesis. Sadahiro and Leda proposed *in vivo* reprogramming as a novel approach to cardiac regeneration therapy (126). Direct reprogramming or transdifferentiation into cardiomyocytes was first demonstrated by Leda et al., who screened 14 transcription factors involved in cardiac development and identified the core cardiac reprogramming transcription factors: GATA binding protein 4 (GATA4), Myocyte enhancer factor 2C (MEF2C), and T-Box Transcription Factor 5 (TBX5), collectively referred to as GMT (127, 128).

In inflammatory vascular endothelial cells (VECs), metabolic changes are characterized by increased glycolysis. Mechanical low shear stress activates hypoxia-inducible factor 1α (HIF-1α) in cultured VECs through the nuclear factor-kB (NF-kB) pathway, leading to the upregulation of OTU deubiquitinating enzyme 7B (Cezanne). HIF-1α increases inflammatory factor production in VECs by upregulating the expression of glycolysis-related regulators, including hexokinase 2 (HK2), glucose transporter 1 (GLUT1), fructose-2,6-biphosphatase 3 (PFKFB3), enolase 2 (ENO2), and the extracellular acidification rate (ECAR), which serves as a direct indicator of glycolytic activity) (36, 129). Metabolic reprogramming appears to exhibit disease specificity: (1) In atherosclerosis, glycolysis and glutaminolysis are increased, while fatty acid beta-oxidation (FAO) and nitric oxide are decreased, leading to EndMT and VEC inflammation; (2) In diabetic angiopathy, glycolysis decreases, resulting in an accumulation of glycolysis intermediates; (3) In tumor neovascularization, both glycolysis and oxidative phosphorylation increase, leading to VEC hyperproliferation; (4) In pulmonary arterial hypertension, glycolysis increases while FAO and nitric oxide decrease, resulting in VEC hyperproliferation and dysfunction; and (5) In hypertension, xanthine oxidase increases, while nitric oxide decreases, causing oxidative stress (36). However, it remains unclear whether these pathological metabolic reprogramming events lead to pathological transdifferentiation. Several metabolic pathways involved in VSMCs transition from contractile to proliferative states in coronary artery disease have been identified, including nitrogen compound metabolism, cellular macromolecule biosynthesis, primary metabolic processes, and the positive regulation of nitrogen compound metabolism (130).

Protein and histone acylation, a crucial type of post-translational modifications, plays a role in various physiological processes (98), including cell differentiation and energy metabolism (131). Recently, fifteen types of short-chain lysine (Lys) acylation have been identified on histones. These include Lys propionylation, Lys butyrylation, Lys 2-hydroxyisobutyrylation, Lys succinylation, Lys malonylation, Lys glutarylation, Lys crotonylation, Lys β-hydroxybutyrylation (132), Lysine acetylation, Lys lactylation, and 2-hydroxyisobutyrylation (133), formylation, palmitoylation, myristoylation, and benzoylation (131). Research indicates that these modifications influence gene expression and are both structurally and functionally distinct from the well-explored histone Lysine methylation, phosphorylation, small ubiquitin-related modifier (SUMO)ylation, and ubiquitinylation as we reported for chronic metabolic disease-driven 164 histone modification enzyme expressions (98) including regulatory enzymes (writers, readers, and erasers) (131). Similar to our findings on trained immunity (innate immune memory and inflammation enhancement) (50, 80, 86, 134, 135), increased glycolysis (48, 49, 102), increased acetyl-CoA generation and increased mevalonate-cholesterol biosynthesis (49, 102, 136), increased S-adenosylhomocysteine (SAH) (136), and increased bioenergistic activities (137), metabolites-facilitated histone modifications underlie epigenetic memory (138) for pathological transdifferentiation. In chronic inflammation, metabolites-modified histones regulate gene expression, while oncometabolites promote tumorigenesis by increasing protein acylation, which results in chromosomal remodeling and modifications of non-histone protein (133).

Tan, Y et al. demonstrate the therapeutic benefits of metabolic reprogramming in improving cancer chemotherapy. Utilizing high-throughput stimulated Raman scattering imaging and single-cell analysis, they found that cells resistant to cisplatin show heightened uptake of fatty acids (FA), while exhibiting reduced glucose uptake and lipogenesis. This suggests a metabolic reprogramming from glucose-based to FA-dependent anabolic and energy metabolism. Such a shift supports cancer cell survival in the face of oxidative stress induced by cisplatin, priming by boosting fatty acid beta-oxidation (FAO) (139). Inhibiting FAO with a small molecule blocker, when combined with cisplatin or carboplatin, works synergistically to reduce ovarian cancer cell proliferation *in vitro* and to inhibit the growth of patient-derived xenografts *in vivo*. This work supports a method for rapidly detecting cisplatin-resistance at the single-cell level, as well as a strategy for treating tumors that are resistant to cisplatin (140). Mitochondria, long viewed as the cell's “powerhouses” for their essential role in energy production, have now been recognized as multifunctional organelles that connect bioenergetics, immunity, and metabolic signaling. We recently introduced the innovative concept of intracellular immunity and proposed that mitochondria as the leading immune organelles, emphasizing the role of mitochondria as crucial hubs for immune signaling (138). Metabolic reprogramming-offers a promising model for developing new cardiovascular disease therapies not only to inhibit trained immunity but also to suppress pathological transdifferentiation.

## Conclusion

**Figure 1:**
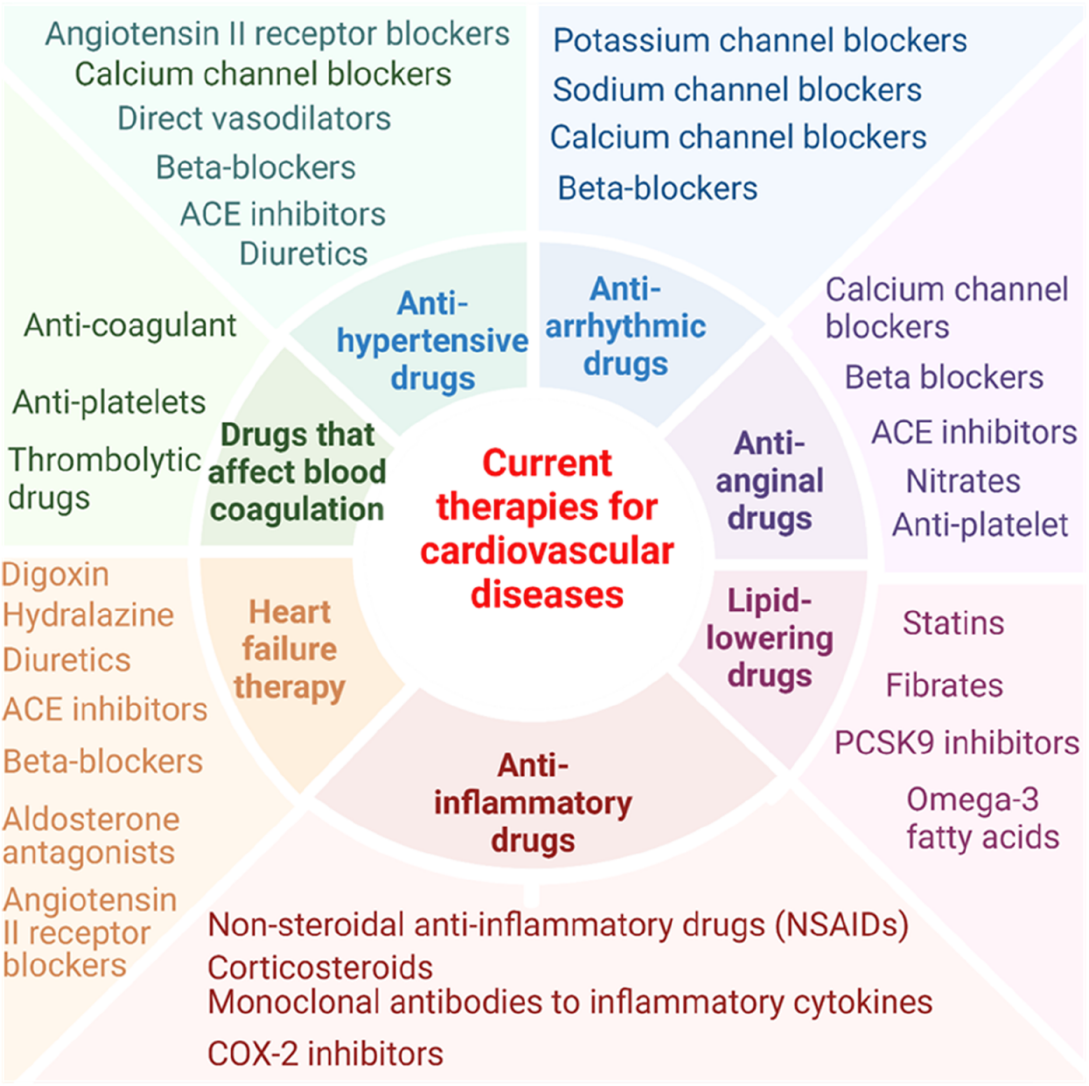
Overview of pharmacological approaches in cardiovascular disease management.

In this perspective, we have analyzed recent progress in characterizing pathological transdifferentiation across various cardiovascular diseases, highlighting the underlying molecular mechanisms and exploring potential new therapeutics targeting this process. Of note, it has been reported that nonsteroidal anti-inflammatory drugs have cardiovascular risk (141) and anti-inflammatory drug glucocorticoids are also found with increasing cardiovascular risk (142). Current therapies for cardiovascular diseases tend to focus on single molecular target (143) (Figure 1). For example, the cholesterol-lowering drug statins inhibit 3-hydroxy-3-methylglutaryl-CoA (HMG-CoA) reductase (144); the anti-inflammatory and anti-atherosclerosis monoclonal antibody (Mab) drug Canakinumab targets interleukin-1β (IL-1β) (86, 137, 145); and the fully human anti-PD-1 monoclonal antibody MEDI0680 inhibits the prototypic immune checkpoint programmed cell death protein 1 (PD-1) (146). Pathological transdifferentiation represents a new therapeutic target that could prevent chronic inflammation and cardiovascular disease-induced cell death (113), block tissue injury-triggered pathological transdifferentiation and restore cellular and tissue functions. Tissue remodeling is indeed a complex and sometimes poorly defined therapeutic target in various diseases due to the complexity of the remodeling process, lack of biomarkers, and the variability across diseases (147–149). Pathological transdifferentiation provides a more defined and targeted approach compared to the broader and more complex concept of tissue remodeling. With ongoing research and advancements in technology, regenerative transdifferentiation (restore tissue function) holds significant promise for regenerative medicine and therapeutic interventions across various diseases.

**Figure 2:**
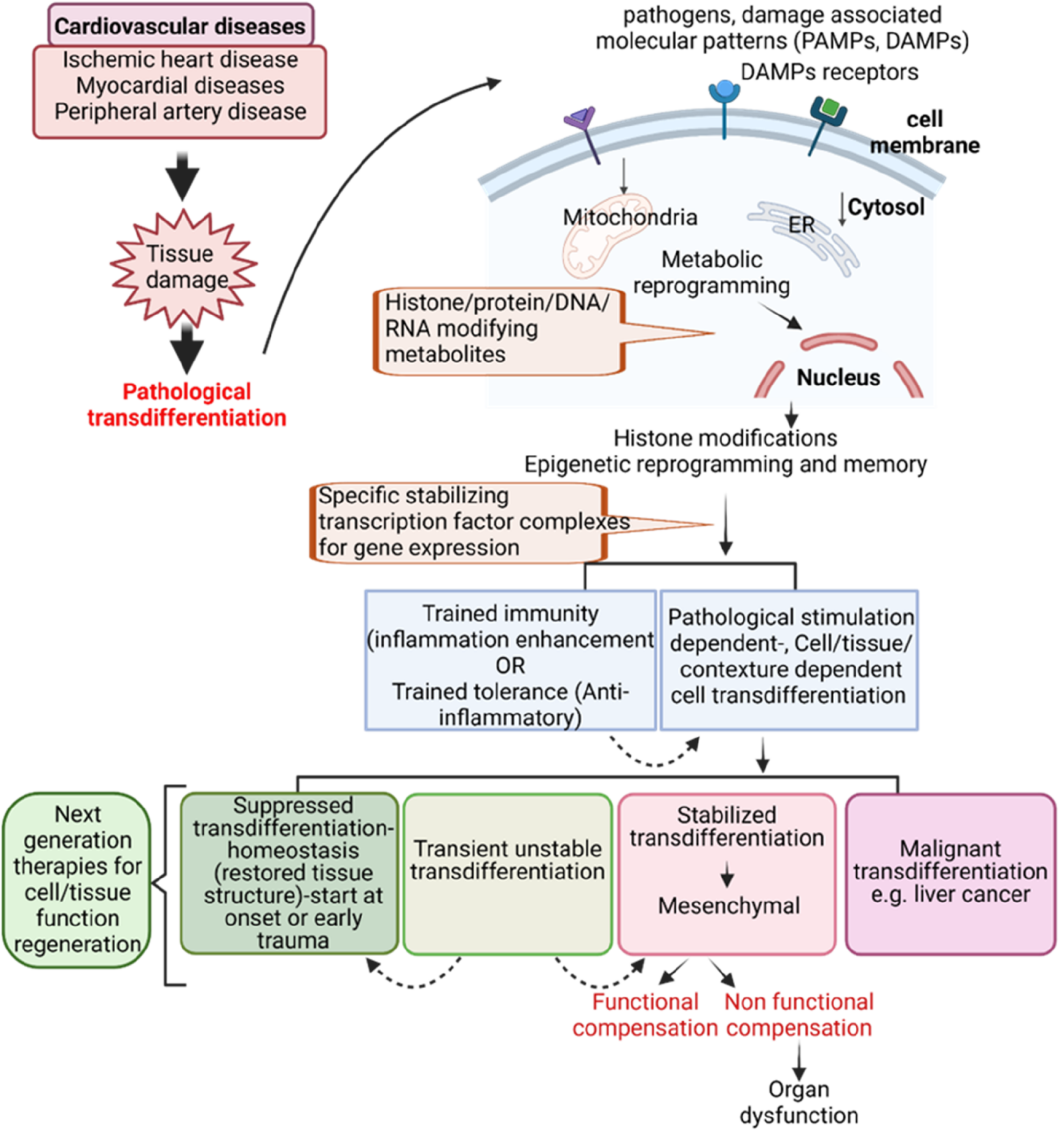
Innovative model for targeting pathological transdifferentiation in cardiovascular disease treatment.

To address this perspective, we propose a new working model (Figure 2): (1) cardiovascular diseases such as myocardial diseases, ischemic heart diseases, and peripheral arterial diseases (150) can induce tissue damage and fibrosis leading to pathological trandifferentiation. Chronic inflammation and cardiovascular disease risk factors generate a variety of DAMPs, PAMPs, cytokines and chemokines, secretomes, and cell death receptors. These pathological signals activate intracellular signaling cascades that lead to the activation of cell lineage-specific differentiation and transdifferentiation transcription factors, including oncogenic transcription factors (oncogenesis, outside the scope of this perspective). This process drives transcriptomic reprogramming, which underpins pathological transdifferentiation. To establish the pathological transdifferentiation program, these signals trigger different forms of metabolic reprogramming, including disease-specific, disease stage-specific, pathological cell type-specific, cell origin-specific, and tissue specific metabolic reprogramming. These metabolic reprogramming generate unique sets of metabolites or combinations of metabolites, which in turn drive specific epigenetic and histone modifications. These modifications help to stabilize and epigenetically memorizing the pathological transdifferentiation processes driven by the specific transcription factors. The concept of metabolic reprogramming leading to epigenetic memory has also been reported in studies of trained immunity (also known as innate immune memory) and trained tolerance (22, 24, 48–50, 82, 102, 134, 135, 151). This new working model enhances our understanding of chronic inflammation- and cardiovascular disease-induced pathological transdifferentiation and identifies new therapeutic targets for treating these diseases, as well as for regenerative medicine.

## Data Availability

The original contributions presented in the study are included in the article/Supplementary Material, further inquiries can be directed to the corresponding author.
